# Association Between Health Literacy and Subjective Health Perception: Analysis of a National Survey in Korea

**DOI:** 10.3390/healthcare14101353

**Published:** 2026-05-15

**Authors:** Se Ryeon Lee, Eun-Yeob Kim, Chilhwan Oh, Jaeyoung Kim

**Affiliations:** 1 Division of Oncology/Hematology, Department of Internal Medicine, Korea University Ansan Hospital, Ansan 15355, Republic of Korea; logost@korea.ac.kr; 2 Research Institute for Skin Image, Korea University College of Medicine, Seoul 08308, Republic of Korea; key0227@korea.ac.kr; 3 School of Medicine and Hospital, Wonkwang University, Iksan 54538, Republic of Korea; choh@korea.ac.kr; 4 Department of Convergence medicine, College of Medicine, Korea University, Seoul 02841, Republic of Korea; 5 Core Research & Development Center, Korea University Ansan Hospital, Ansan 15355, Republic of Korea

**Keywords:** health literacy, health information, information-seeking behavior, health behavior, subjective health perception

## Abstract

**Background/objectives:** With the rapid expansion of Internet-based health information, individuals increasingly rely on digital sources to obtain medical knowledge and manage their health. Health literacy plays a critical role in determining how effectively individuals access, understand, and utilize such information. This study aimed to examine the association between subjective health perception and health information literacy-related indicators among Korean adults. **Methods**: This cross-sectional study utilized secondary data from the 2019 “Health Information Literacy Improvement Study” conducted by the Korea Institute for Health and Social Affairs. A total of 1000 adults aged 19–69 years were included in the analysis. Participants were categorized into three groups according to subjective health perception (good, normal, and poor). Descriptive statistics and chi-square tests were conducted to examine differences. In addition, multinomial logistic regression analysis was performed to identify factors associated with subjective health perception. **Results**: Participants with better subjective health perception reported fewer chronic diseases (*p* < 0.001), healthier dietary behaviors (*p* < 0.001), and more frequent health information seeking (*p* = 0.023). They also reported greater ease in finding and understanding health information (*p* < 0.001). Multinomial logistic regression analysis revealed that health information literacy-related factors, including information-seeking behavior and the ability to evaluate information reliability, were significantly associated with subjective health perception. Individuals with fewer chronic diseases and healthier behaviors were less likely to report poor subjective health. **Conclusions**: Subjective health perception was significantly associated with multiple health information literacy-related indicators and health-seeking behaviors. These findings highlight the importance of improving health information literacy-related competencies to support informed health decision-making and promote positive health perceptions.

## 1. Introduction

With the advent of the Internet, significant changes have occurred in society. In 2018, 91.5% of Korean citizens used the Internet, marking a 1.2% increase from the previous year, and this figure continues to rise with the widespread adoption of smartphones [1,2]. This digital transformation has increasingly positioned the Internet as a crucial source of personal health information [3]. In the fields of health and medicine, the Internet has catalyzed changes at all societal levels, including governments, communities, individuals, and organizations [4,5]. Unlike in the pre-Internet era, health information available to the public has become abundant [6]. An individual’s ability to effectively use Internet health information or smartphones significantly impacts their health information literacy, also known as health literacy [7,8].

Today, health information is readily accessible from various sources, including government websites, private medical sites, and online community social networks via the Internet and smartphones [9]. The number of users actively seeking and utilizing health information through these digital platforms is steadily increasing [10,11]. The ability to accurately locate and interpret high-quality information amidst the vast amounts available is a critical issue in the current information age [12]. In response to this reality, the concept of “e-health literacy” has emerged. It refers to the ability to search for, understand, evaluate, and effectively use health information found on the Internet to address and solve health-related problems [13,14,15]. The importance of applying and conveying the acquired knowledge has also gained prominence [13,14]. Concurrently, health literacy, defined as the ability to comprehend and apply health information, has become a significant public health issue that healthcare systems must address [16,17,18]. Health literacy not only affects individuals’ ability to obtain and process health information but also plays a crucial role in shaping their cognitive and perceptual evaluation of their own health status. In this context, subjective health perception—defined as an individual’s self-assessment of their overall health—has emerged as an important indicator reflecting both physical and psychological health conditions [19,20,21]. Health literacy is related to subjective health perception—how individuals perceive their own health—which is a key determinant of actual health outcomes and healthcare utilization [19].

Previous studies have demonstrated that individuals with higher health literacy are more likely to report better subjective health status, engage in preventive health behaviors, and utilize healthcare services appropriately [20,21,22,23]. Conversely, limited health literacy has been associated with poorer health perceptions, inadequate health behaviors, and increased health disparities [15,23]. Despite these findings, most prior research has primarily focused on objective health outcomes or healthcare utilization, with relatively limited attention given to the direct relationship between health literacy and subjective health perception, particularly within the context of rapidly expanding digital health environments [15,22].

Furthermore, although e-health literacy has received increasing attention in recent years, there remains a lack of comprehensive research examining how the use of digital health information interacts with traditional health literacy to influence individuals’ subjective health awareness [24,25,26,27]. In particular, studies focusing on general adult populations that simultaneously consider sociodemographic characteristics, health-related factors, and subjective health perception are still insufficient [21,24]. This gap underscores the need for an integrated approach to better understand how health literacy shapes individuals’ perceptions of their own health in the modern digital context.

This study aimed to examine health literacy levels in adults, focusing on their subjective health awareness and the influencing sociodemographic and health-related characteristics. The findings highlight the association between subjective health status and health-seeking behavior.

## 2. Materials and Methods

### 2.1. Study Design and Variables

This study utilized data from the 2019 research project titled “Ways to Improve Health Information Literacy,” conducted by the Korea Institute for Health and Social Affairs under the Ministry of Health and Welfare. Secondary open data were utilized to assess the health information literacy of 1000 Korean citizens (492 women and 508 men; average age: 44 years, range: 19–69 years). Participants’ subjective health perceptions were categorized into three groups: good, normal, and poor. This study employed a cross-sectional design utilizing nationally collected survey data. All variables were derived from structured, self-administered questionnaires and analyzed as self-reported measures.

In this study, health literacy was operationally defined as the ability to search for, understand, evaluate, and utilize health-related information obtained through digital and traditional sources. Because the original dataset did not include a validated health literacy scale (e.g., HLS-EU or REALM), proxy indicators were used to assess health information literacy. These indicators included frequency of health information searching, perceived effort required to obtain health information, perceived ease of understanding health information, and perceived importance of health information. Specifically, health information literacy was assessed using multiple self-reported items, which were conceptually categorized into domains such as information-seeking behavior, accessibility, comprehension, and appraisal/utilization. These domains were not based on a validated scale but were derived from the characteristics of the survey items included in the dataset. Each item was measured using ordinal or Likert-type response scales (e.g., “strongly disagree” to “strongly agree” or “very difficult” to “very easy”).

The study analyzed the following general variables: gender, age, area of residence, smoking status (including conventional and electronic cigarettes), alcohol consumption, number of chronic diseases, healthy eating habits, nutrition label usage, healthcare utilization, unmet medical needs, household size, marital status, education level, occupation, household economic status, and monthly income. These variables were measured as either categorical or continuous based on self-reported responses. For example, smoking status was classified as current, occasional, or non-smoker; alcohol consumption was categorized by frequency; and the number of chronic diseases was grouped into none, one, two, or three or more. Healthcare utilization variables included the frequency of visits to medical institutions and experiences of unmet medical needs. Socioeconomic variables, such as education, occupation, and income, were categorized into multiple predefined levels. Health information literacy-related variables included the following items, all of which were directly obtained from questionnaire responses and analyzed as categorical or ordinal variables: Information-Seeking Behavior—Frequency of Searching for Health and Medical Information Accessibility: This variable refers to the perceived effort required to locate information and the inconvenience experienced during the information-seeking process. Perceived Importance of Health Information: This refers to the significance of up-to-date information, reliability, ease of understanding, official sources, comprehensiveness, and diversity of perspectives. Comprehension and Understanding: This refers to the ability to grasp doctors’ explanations, medication instructions, and health information obtained from the media or acquaintances. Appraisal and Decision-Making: This is the ability to assess the reliability of health information, determine when a second opinion is necessary, and make informed treatment decisions based on medical data. Application/Utilization: This refers to the ability to follow medical instructions, manage mental health, recognize warning signs of health risks, understand the importance of regular health checkups, and evaluate the relationship between lifestyle habits and overall health. Outcome Variable: Subjective health perception, the primary outcome variable, was assessed using a single-item self-rated health question and categorized into three levels: good, fair, and poor.

### 2.2. Statistical Analysis

For statistical analysis, a database was created using Microsoft Excel. Descriptive statistics were used to summarize participant characteristics, and group differences were analyzed using chi-square tests or the Kruskal–Wallis test, depending on the variable characteristics. Categorical variables were analyzed using chi-square tests, while ordinal variables were analyzed using non-parametric methods. Continuous variables (e.g., age) are expressed as mean ± standard deviation and analyzed using appropriate comparative tests. All analyses were conducted by comparing groups according to subjective health perception (good, normal, and poor). To identify factors associated with subjective health perception, multinomial logistic regression analysis was conducted using subjective health perception (categorized as good, normal, or poor) as the dependent variable, with the “good” category serving as the reference group. Predictor variables were selected based on theoretical relevance and significant results from bivariate analyses, while avoiding multicollinearity among conceptually overlapping variables. The final model included sex, frequency of health information seeking, difficulty in accessing health information, comprehension of doctors’ explanations, ability to evaluate the reliability of health-related media information, chronic disease status, healthy eating habits, and educational level. Ordinal variables measured using Likert-type scales were treated as categorical variables in the regression analysis. Model results were assessed for unstable parameter estimates and excessively wide confidence intervals (Cis). Categories exhibiting unstable estimates due to sparse data were interpreted cautiously and excluded from substantive interpretation when appropriate. Adjusted odds ratios (ORs) and 95% CIs were reported. The analyses were conducted using IBM SPSS Statistics for Windows, version 25.0 (IBM Corp., Armonk, NY, USA), with statistical significance set at *p* < 0.05.

## 3. Results

### 3.1. Subjective Health Perception and General Characteristics

The results concerning subjective health perception and general characteristics are presented in Table 1. Although men tended to report better subjective health perception than women, the difference did not reach statistical significance (*p* = 0.079). Younger participants tended to report better subjective health perception; however, this difference was not statistically significant (*p* = 0.166). Better subjective health perception was associated with fewer chronic diseases (*p* < 0.001). For conventional cigarettes, individuals with poorer subjective health perception showed lower smoking cessation rates, but with no significant difference between the groups (*p* = 0.062). In contrast, for e-cigarettes, a better subjective health perception was linked to a higher smoking cessation rate (*p* = 0.006). Individuals with a better subjective health perception were more likely to practice healthy eating habits (*p* < 0.001) and to check nutrition labels when purchasing processed foods (*p* < 0.001). The frequency of visits to medical institutions for treatment increased with worsening subjective health perception, exceeding three times per month (*p* < 0.001). Conversely, individuals with poor subjective health perceptions more frequently reported needing medical treatment at a hospital or clinic but not receiving it (*p* = 0.005). In terms of educational background, all groups had at least a high school education, but those with a positive subjective health perception were more likely to have completed graduate school or higher education (*p* = 0.005). Regarding occupations, individuals with positive subjective health perceptions were more likely to be wage earners, whereas those with normal or poor health perceptions were more likely to be non-economically active (*p* < 0.001). Individuals with a better subjective health perception reported above-normal family circumstances (*p* < 0.001) and were more likely to have an income higher than 4 million won (*p* < 0.001) (Table 1).

### 3.2. Accessibility of Health/Medical Information According to Subjective Health Perception

Accessibility-related findings are presented. Individuals with a better subjective perception of their health reported more frequent health information seeking (*p* = 0.023) and experienced less inconvenience in accessing such information (*p* < 0.001).

### 3.3. Health Information Literacy–Related Indicators According to Subjective Health Perception

The results concerning the perceived importance of searching for health/medical information are presented. Across all three groups with varying levels of subjective health perception, participants consistently rated aspects such as the latest health information, reliable sources, quickly understanding key points, information from official sources, comprehensiveness, and diverse opinions as “important.” However, the groups showed no differences in these aspects. Significant differences were observed among the groups regarding specific aspects of health/medical information. Individuals with a better subjective perception of their health were more likely to find it easier to locate treatment information for specific concerns, seek out a medical specialist or specialized hospital when necessary, understand explanations provided by doctors during treatment, comprehend instructions from doctors or pharmacists regarding prescriptions, determine when to seek a second opinion, use information provided by doctors to make treatment decisions, find information on managing mental health issues, recognize warning symptoms of health-threatening behavior, understand the importance of health checkups, assess the reliability of health-related media information, decide on health management strategies using media information, find physical activities beneficial for mental health, understand health information provided by family members, and comprehend health promotion information through media (all *p* < 0.001). They were also more likely to follow health management or medication instructions (*p* = 0.003) and study the correlation between daily lifestyle habits and health (*p* = 0.002).

In addition, the frequency of searching for health/medical information more than once a week to almost daily was higher among those with a better subjective health perception (*p* = 0.023). They also reported experiencing less discomfort in finding health/medical information (*p* < 0.001). Although individuals with a better subjective health perception tended to exert less effort in finding health/medical information, the difference was not significant (*p* = 0.097) (Table 2).

### 3.4. Factors Associated with Subjective Health Perception

Multinomial logistic regression analysis revealed that several factors were significantly associated with subjective health perception. Male participants were less likely to report poorer subjective health compared to female participants. Individuals who searched for health information less frequently were less likely to report moderate health status compared to good health. Participants who experienced less inconvenience in accessing health information were less likely to belong to the poor health group. In contrast, a higher ability to judge the reliability of health-related information was strongly associated with both moderate and good subjective health perceptions. Regarding health status, individuals with fewer chronic diseases were significantly less likely to report poor subjective health. Healthy eating habits were also positively associated with better subjective health perception. Educational variables showed unstable estimates and were therefore excluded from the final interpretation (Table 3).

## 4. Discussion

This study examined differences in health information literacy according to levels of subjective health perception and assessed the association between subjective health perception and health literacy. Previous research has identified factors such as age, income, education, and gender as influencing factors for health information literacy. Urban residents and individuals with higher incomes tend to show lower engagement in seeking health information and lower efficacy in utilizing it [24,25,26]. Neter and Brainin found a correlation between high levels of e-health literacy and younger age and higher education levels among Israeli adults aged ≥18 years [15]. Consistent with these findings, our study also observed that better subjective health perception was associated with higher educational attainment and younger age. Moreover, individuals with better subjective health perception tended to have fewer chronic diseases, indicating a higher level of health awareness and engagement in health-related behaviors. Furthermore, our results indicated that better subjective health perception was associated with higher income, greater educational attainment, favorable family circumstances, healthy eating habits, diligent checking of processed food information, and being in a marital relationship. These findings suggest that a better subjective health perception is associated with an increased interest in health-related information and a greater motivation to adopt health-promoting behaviors [26,27]. These associations may reflect differences in health-related behaviors and access to resources rather than indicating causal relationships. While previous studies have primarily focused on demographic and socioeconomic determinants of health literacy, this study offers an integrated perspective by examining subjective health perception alongside multiple dimensions of health information literacy. Notably, the findings suggest that subjective health perception is not merely an outcome but is closely linked to individuals’ engagement with health information. This extends the existing literature by highlighting the potential bidirectional relationship between health perception and health information literacy.

Regarding the accessibility of health/medical information, individuals with better subjective health awareness reported less difficulty in finding or understanding health information. They tended to search for information more frequently, often on a weekly or daily basis. These findings suggest that individuals with better subjective health perceptions are more actively engaged in health information-seeking behaviors. In addition, better subjective health perception was linked to higher income levels and favorable family circumstances. These results underscore the importance of subjective health awareness in relation to individuals’ engagement with health-related information and their ability to utilize it effectively. Shim highlighted that the use of e-health information and subsequent changes in health behaviors vary depending on perceived health status, which is influenced by intentional engagement with e-health information and the timing of information utilization [22]. Our findings support this notion, indicating that individuals with higher levels of subjective health awareness encounter fewer challenges in accessing medical information and require less effort to search for it.

In addition, the importance of seeking health/medical information was rated across three subjective health awareness groups, with the latest information, authoritative sources, rapid comprehension of critical information, official sources, comprehensive information, and diverse opinions rated as “important” or higher. Moreover, individuals with a more positive health perception were more likely to easily access specific treatment information for their conditions, find medical experts for assistance, and comprehend medical explanations and prescriptions. This finding suggests that individuals who perceive their health more positively tend to engage more actively in health information processing and utilization [22].

Further, Knapp found that individuals who do not speak English and older adults with lower educational attainment have lower levels of e-health literacy [25,26]. In our study, improved subjective health awareness was associated with easier decision-making regarding health management or medication adherence, accessing information on mental health management, understanding warning signs of health-related behaviors, recognizing the importance of health checkups, evaluating the reliability of health information in the media, and using media for health-related decision-making. Furthermore, individuals with higher subjective health awareness find it easier to understand health information from family members and health promotion messages through media channels [22,25,26]. These findings highlight the importance of subjective health awareness as a factor associated with differences in health-related behaviors and information-seeking practices. Interestingly, while prior research has often emphasized structural factors such as education and income, the present findings suggest that cognitive and perceptual factors—such as subjective health awareness—may play a more prominent role in shaping health information behaviors. This finding indicates a potential shift from structural determinants toward individual-level cognitive engagement in understanding health literacy.

As a result, increased awareness and interest in one’s health may be associated with a more proactive approach to understanding the importance of health and medical information in developing healthy lifestyles and habits. Moreover, individuals with a strong subjective perception of their health may be better positioned to make informed judgments and decisions regarding their well-being. This positive self-perception is associated with broader health-related perceptions, laying a foundation to mitigate negative outcomes, such as depression and suicide. By consistently prioritizing self-care and health management, individuals may enhance their overall health and foster positive health-seeking behaviors. The present study’s findings support the hypothesis that higher levels of health information literacy-related indicators are associated with better subjective health perceptions. Individuals with higher levels of health information literacy-related competencies are more likely to understand health information, make informed health decisions, and engage in health-promoting behaviors. These factors contribute to a more positive perception of their health.

In light of these findings, interventions aimed at enhancing both health literacy and subjective health status may be beneficial for fostering a proactive approach to health. Education and informational guidance are key to increasing interest in health and promoting positive health attitudes among individuals. Tailored health programs should be developed based on the characteristics of populations with lower subjective health awareness to effectively encourage health-seeking behaviors.

Furthermore, reliable and accurate dissemination of health information is needed to comprehensively address health concerns. In this information age, collaboration among healthcare providers, medical institutions, and health education organizations is essential to enhance health information literacy and encourage health-seeking behaviors. Future longitudinal studies are warranted to further explore the dynamics among subjective health awareness, health information literacy, and health behaviors. Continuous evaluation and refinement of health promotion strategies will contribute to improving public health outcomes and fostering a healthier society.

This study has several limitations. Due to its cross-sectional design, the findings should be interpreted as associations rather than causal relationships. First, the study relied on secondary survey data from a national research project of 1000 people, based on a survey by a domestic national agency, making it insufficient to represent health literacy for specific population groups. Second, we could not accurately assess the appropriateness of the health information obtained by individuals; thus, future research should provide guidelines for determining the quality of health information. Nevertheless, the results of this study are expected to contribute to validated health literacy research within the country. Third, this study used proxy indicators to assess health information literacy because a validated measurement instrument (e.g., HLS-EU or eHEALS) was not available in the secondary dataset. Although these proxy variables were chosen to reflect key dimensions of health information literacy—such as information seeking, accessibility, comprehension, and evaluation—they may not fully capture the construct’s complexity. Therefore, caution is warranted when interpreting the findings, as the construct validity may be limited. Future research employing standardized and validated instruments is necessary to confirm these results.

Because this study was based on a cross-sectional design, causal relationships between health literacy and subjective health perception cannot be established; therefore, the findings should be interpreted as associations. It is possible that individuals with higher health literacy perceive their health more positively. Conversely, individuals who perceive themselves as healthy may be more motivated to seek and utilize health information. Therefore, the relationship between these variables may be bidirectional. The data used in this study were collected in 2019, prior to the COVID-19 pandemic. Since the pandemic has significantly increased the public’s reliance on digital health information, health information-seeking behaviors may have changed substantially. Therefore, caution is required when generalizing these findings to the current post-pandemic environment.

Improving health information literacy-related competencies may play an important role in promoting proactive health behaviors. Public health strategies should therefore focus on enhancing access to reliable health information and improving individuals’ ability to critically evaluate health information obtained from digital sources. As digital health information continues to expand, the ability to critically evaluate and utilize health information has become an essential competency for modern healthcare consumers. Strengthening health information literacy-related competencies may represent a key strategy for improving population health outcomes.

From a practical perspective, these findings suggest that interventions aimed at improving health information literacy-related competencies should extend beyond merely providing information to also address individuals’ subjective perceptions of their health. Tailored strategies that enhance both awareness and engagement are likely to be more effective in promoting health-seeking behaviors. Furthermore, public health policies should incorporate approaches that support individuals in critically evaluating and effectively utilizing health information in digital environments.

## 5. Conclusions

This study investigated the relationship between subjective health awareness, health information literacy-related indicators (specifically medical information), and health-seeking behavior, identifying associations between subjective health status and these domains. The findings highlight a positive correlation between subjective health awareness and health information literacy-related indicators, indicating that subjective health status is associated with health-seeking behavior. In addition, the role of health information literacy-related indicators in relation to subjective health perception was demonstrated in this study. Individuals with higher levels of health information literacy-related indicators reported better perceived health, which may be related to their overall well-being and healthcare behaviors. Because the study used proxy indicators instead of a validated health literacy instrument, the findings should be interpreted with caution. These findings contribute to the growing body of literature by highlighting the association between subjective health perception, health information literacy indicators, and health-related behaviors.

## Figures and Tables

**Table 1:** General characteristics of participants according to subjective health perception.

Variables		Good		Normal		Poor		*p*-Value
		N	%	N	%	N	%	
Sex	Men	158	56.2	275	49.4	75	46.3	5.082 (0.079)
	Women	123	43.8	282	50.6	87	53.7	
Age *		43.15 ± 14.01		44.68 ± 12.53		45.29 ± 12.86		1.797 (0.166)
Residence	City	151	53.5	300	53.8	80	49.4	5.152 (0.272)
	Province	107	37.9	222	39.8	75	46.3	
	District	24	8.5	36	6.5	7	4.3	
Chronic disease	No	207	73.4	311	55.7	38	23.5	187.845 (<0.001)
	Yes = 1	68	24.1	199	35.7	62	38.3	
	Yes = 2	5	1.8	44	7.9	46	28.4	
	Yes ≥ 3	2	0.7	4	0.7	16	9.9	
Smoke (cigarette type)	Currently	53	53.0	125	57.6	34	54.0	8.974 (0.062)
	Sometimes	20	20.0	24	11.1	4	6.3	
	Never or no smoking	27	27.0	68	31.3	25	39.7	
Smoke (electronic type)	Yes	50	17.7	60	10.8	15	9.3	10.184 (0.006)
	No	232	82.3	498	89.2	147	90.7	
Drink	Never	21	7.4	33	5.9	14	8.6	16.668 (0.163)
	Not/1 year ago	30	10.6	91	16.3	34	21.0	
	Less than 1 time/a month	54	19.1	118	21.1	34	21.0	
	1 time a month	40	14.2	66	11.8	14	8.6	
	2–4 times a month	75	26.6	134	24.0	35	21.6	
	2–3 times a week	48	17.0	95	17.0	21	13.0	
	more than 4 times a week	14	5.0	21	3.8	10	6.2	
Healthy eating habits	No effort at all	1	0.4	5	0.9	6	3.7	92.742 (<0.001)
	Not to try hard	23	8.2	67	12.0	42	25.9	
	Normal	81	28.7	281	50.4	53	32.7	
	Make an effort	155	55.0	185	33.2	53	32.7	
	Very hard	22	7.8	20	3.6	8	4.9	
Check nutrition labels when purchasing processed foods	No effort at all	16	5.7	40	7.2	22	13.6	41.627 (<0.001)
	Not to try hard	68	24.1	157	28.1	50	30.9	
	Normal	71	25.2	209	37.5	44	27.2	
	Make an effort	103	36.5	129	23.1	36	22.2	
	Very hard	24	8.5	23	4.1	10	6.2	
Visit to a medical institution for treatment/2019 year	More than once a week	5	1.8	6	1.1	6	3.7	35.988 (<0.001)
	2–3 times a month	13	4.6	30	5.4	17	10.5	
	Once a month	43	15.2	63	11.3	32	19.8	
	Once every 2–3 months	60	21.3	152	27.2	51	31.5	
	About once in 6 months	102	36.2	196	35.1	32	19.8	
	Not visit	59	20.9	111	19.9	24	14.8	
Experience of needing medical treatment/2019 year	Yes	17	6.0	56	10.0	27	16.7	14.894 (0.005)
	No	209	74.1	410	73.5	114	70.4	
	Hospital treatment	56	19.9	92	16.5	21	13.0	
Number of family members living together	1	31	11.0	66	11.8	26	16.0	19.333 (0.252)
	2	49	17.4	106	19.0	36	22.2	
	3	85	30.1	156	28.0	40	24.7	
	4	92	32.6	190	34.1	52	32.1	
	5	21	7.4	29	5.2	7	4.3	
	6	3	1.1	9	1.6	0	0.0	
	7	0	0.0	0	0.0	1	0.6	
	8	0	0.0	2	0.4	0	0.0	
	9	1	0.4	0	0.0	0	0.0	
Marital Status	Single	113	40.1	203	36.4	70	43.2	11.174 (0.083)
	A spouse and live together	158	56.0	322	57.7	76	46.9	
	A spouse but do not live together	3	1.1	5	0.9	3	1.9	
	Divorced, separated	8	2.8	28	5.0	13	8.0	
Education	No education	0	0.0	0	0.0	1	0.6	24.984 (0.005)
	Elementary school	0	0.0	2	0.4	1	0.6	
	Middle school	2	0.7	2	0.4	5	3.1	
	High school	51	18.1	125	22.4	43	26.5	
	University/College	193	68.4	379	67.9	98	60.5	
	Higher than college	36	12.8	50	9.0	14	8.6	
Work	Wage workers (including part-time workers)	174	61.7	328	58.8	86	53.1	35.749 (<0.001)
	Employer, self-employed	27	9.6	52	9.3	12	7.4	
	Working unpaid at a family-run business for more than 18 h a week	4	1.4	8	1.4	3	1.9	
	Housewife	39	13.8	88	15.8	25	15.4	
	Student	21	7.4	24	4.3	4	2.5	
	Not engaged in economic activity (retired, preparing for higher education or employment, no intention to work, etc.)	13	4.6	55	9.9	32	19.8	
	Other	4	1.4	3	0.5	0	0.0	
Household level	Lowest	6	2.1	18	3.2	13	8.0	123.033 (<0.001)
	2	11	3.9	32	5.7	18	11.1	
	3	23	8.2	99	17.7	47	29.0	
	4	38	13.5	115	20.6	30	18.5	
	Middle	67	23.8	152	27.2	29	17.9	
	6	65	23.0	94	16.8	15	9.3	
	7	45	16.0	37	6.6	7	4.3	
	8	16	5.7	9	1.6	3	1.9	
	9	5	1.8	1	0.2	0	0.0	
	Highest	6	2.1	1	0.2	0	0.0	
Income	<1 million won	4	1.4	21	3.8	12	7.4	50.729 (<0.001)
	1.0–1.9 million won	18	6.4	39	7.0	17	10.5	
	2.0–2.9 million won	28	9.9	91	16.3	28	17.3	
	3.0–3.9 million won	42	14.9	102	18.3	26	16.0	
	4.0–4.9 million won	46	16.3	108	19.4	20	12.3	
	5.0–5.9 million won	44	15.6	58	10.4	26	16.0	
	6.0–6.9 million won	22	7.8	47	8.4	10	6.2	
	7.0–7.9 million won	25	8.9	34	6.1	11	6.8	
	8.0–8.9 million won	14	5.0	23	4.1	6	3.7	
	9.0–9.9 million won	17	6.0	15	2.7	3	1.9	
	>10.0 million won	22	7.8	20	3.6	3	1.9	

* Age is expressed as mean ± standard deviation.

**Table 2:** Importance of health/medical information according to subjective health perception.

Variables		Good		Normal		Poor		*p*-Value
		N	%	N	%	N	%	
Perceived importance of searching health/medical information								
Latest information	Not important at all	0	0.0	0	0.0	0	0.0	6.218 (0.183)
	Not important	15	5.5	20	3.7	11	6.9	
	Important	165	60.2	361	66.4	94	59.1	
	Very important	94	34.3	163	30.0	54	34.0	
Reliable information	Not important at all	1	0.4	3	0.6	1	0.6	5.143 (0.526)
	Not important	7	2.6	21	3.9	3	1.9	
	Important	91	33.2	195	35.8	47	29.6	
	Very important	175	63.9	325	59.7	108	67.9	
Learn the most important things quickly	Not important at all	1	0.4	3	0.6	2	1.3	9.641 (0.141)
	Not important	28	10.2	60	11.0	15	9.4	
	Important	153	55.8	346	63.6	91	57.2	
	Very important	92	33.6	135	24.8	51	32.1	
Information from official sources	Not important at all	2	0.7	3	0.6	0	0.0	5.169 (0.522)
	Not important	15	5.5	28	5.1	4	2.5	
	Important	123	44.9	265	48.7	72	45.3	
	Very important	134	48.9	248	45.6	83	52.2	
Whether to present a different opinion	Not important at all	2	0.7	3	0.6	1	0.6	3.878 (0.693)
	Not important	37	13.5	89	16.4	19	11.9	
	Important	192	70.1	368	67.6	119	74.8	
	Very important	43	15.7	84	15.4	20	12.6	
Comprehensiveness of information	Not important at all	2	0.7	3	0.6	1	0.6	3.288 (0.772)
	Not important	40	14.6	89	16.4	30	18.9	
	Important	171	62.4	354	65.1	98	61.6	
	Very important	61	22.3	98	18.0	30	18.9	
Perceived ease of finding, understanding, or using health/medical information								
Finding treatment information for the disease you are concerned about	Very difficult	12	4.3	19	3.4	8	4.9	30.333 (<0.001)
	Difficult	104	36.9	277	49.6	92	56.8	
	Easy	145	51.4	248	44.4	59	36.4	
	Very easy	21	7.4	14	2.5	3	1.9	
Finding a medical professional (specialized hospital) to help you when you are sick	Very difficult	24	8.5	32	5.7	21	13.0	51.905 (<0.001)
	Difficult	89	31.6	270	48.4	82	50.6	
	Easy	136	48.2	235	42.1	55	34.0	
	Very easy	33	11.7	21	3.8	4	2.5	
Understand what the doctor explains during treatment	Very difficult	19	6.7	24	4.3	10	6.2	31.043 (<0.001)
	Difficult	99	35.1	284	50.9	85	52.5	
	Easy	142	50.4	232	41.6	65	40.1	
	Very easy	22	7.8	18	3.2	2	1.2	
Understand the doctor or pharmacist’s instructions on how to take prescribed medications	Very difficult	15	5.3	17	3.0	3	1.9	29.369 (<0.001)
	Difficult	51	18.1	170	30.5	56	34.6	
	Easy	171	60.6	325	58.2	86	53.1	
	Very easy	45	16.0	46	8.2	17	10.5	
After receiving treatment from a doctor, determine whether it is necessary to receive treatment from another doctor	Very difficult	29	10.3	53	9.5	16	9.9	37.322 (<0.001)
	Difficult	120	42.6	299	53.6	100	61.7	
	Easy	108	38.3	195	34.9	43	26.5	
	Very easy	25	8.9	11	2.0	3	1.9	
Use the information given by your doctor to make decisions about treatment for your illness	Very difficult	7	2.5	26	4.7	10	6.2	38.812 (<0.001)
	Difficult	99	35.1	259	46.4	86	53.1	
	Easy	148	52.5	253	45.3	65	40.1	
	Very easy	28	9.9	20	3.6	1	0.6	
Follow healthcare and medication instructions as instructed by a doctor or pharmacist	Very difficult	8	2.8	13	2.3	4	2.5	19.437 (0.003)
	Difficult	51	18.1	144	25.8	47	29.0	
	Easy	160	56.7	329	59.0	92	56.8	
	Very easy	63	22.3	72	12.9	19	11.7	
Find information on how to manage mental health issues such as stress or depression	Very difficult	15	5.3	30	5.4	11	6.8	35.838 (<0.001)
	Difficult	89	31.6	265	47.5	88	54.3	
	Easy	150	53.2	239	42.8	57	35.2	
	Very easy	28	9.9	24	4.3	6	3.7	
Understand the warning signs of risky health behaviors such as smoking, lack of exercise, and excessive drinking	Very difficult	16	5.7	16	2.9	10	6.2	34.838 (<0.001)
	Difficult	61	21.6	185	33.2	57	35.2	
	Easy	162	57.4	317	56.8	89	54.9	
	Very easy	43	15.2	40	7.2	6	3.7	
Understand the need for health checkup	Very difficult	6	2.1	6	1.1	7	4.3	37.060 (<0.001)
	Difficult	49	17.4	153	27.4	47	29.0	
	Easy	161	57.1	334	59.9	92	56.8	
	Very easy	66	23.4	65	11.6	16	9.9	
Judging the reliability of media information about health risks	Very difficult	17	6.0	28	5.0	16	9.9	37.022 (<0.001)
	Difficult	109	38.7	294	52.7	95	58.6	
	Easy	135	47.9	221	39.6	48	29.6	
	Very easy	21	7.4	15	2.7	3	1.9	
Decide how to manage your personal health using media information	Very difficult	10	3.5	23	4.1	14	8.6	48.321 (<0.001)
	Difficult	107	37.9	281	50.4	98	60.5	
	Easy	138	48.9	237	42.5	47	29.0	
	Very easy	27	9.6	17	3.0	3	1.9	
Finding physical activities that are good for your mental health	Very difficult	11	3.9	14	2.5	9	5.6	38.185 (<0.001)
	Difficult	84	29.8	222	39.8	76	46.9	
	Easy	146	51.8	290	52.0	72	44.4	
	Very easy	41	14.5	32	5.7	5	3.1	
Understand health information provided by family or friends	Very difficult	6	2.1	13	2.3	4	2.5	33.691 (<0.001)
	Difficult	60	21.3	148	26.5	46	28.4	
	Easy	167	59.2	365	65.4	102	63.0	
	Very easy	49	17.4	32	5.7	10	6.2	
Understand health promotion information provided by the media	Very difficult	9	3.2	12	2.2	1	0.6	37.486 (<0.001)
	Difficult	59	20.9	186	33.3	72	44.4	
	Easy	183	64.9	331	59.3	83	51.2	
	Very easy	31	11.0	29	5.2	6	3.7	
Judging the relationship between daily lifestyle habits and my health	Very difficult	10	3.5	18	3.2	9	5.6	20.990 (0.002)
	Difficult	91	32.3	208	37.3	69	42.6	
	Easy	148	52.5	305	54.7	77	47.5	
	Very easy	33	11.7	27	4.8	7	4.3	
Frequency of searching for health/medical information								
How often do you search for information	Almost everyday	36	12.8	43	7.7	17	10.5	26.349 (0.023)
	More than once a week	89	31.6	145	26.0	44	27.2	
	2–3 times a month	68	24.1	113	20.3	41	25.3	
	Once a month	22	7.8	60	10.8	20	12.3	
	Once every 2–3 months	20	7.1	53	9.5	14	8.6	
	1 time in six months	15	5.3	38	6.8	9	5.6	
	Rarely	24	8.5	92	16.5	14	8.6	
	Never	8	2.8	14	2.5	3	1.9	
Effort and convenience in finding health/medical information								
A lot of effort to find information	Strongly disagree	11	4.0	14	2.6	2	1.3	10.721 (0.097)
	Slightly disagree	87	31.8	158	29.0	38	23.9	
	Slightly agree	147	53.6	330	60.7	99	62.3	
	Strongly agree	29	10.6	42	7.7	20	12.6	
Inconvenience in the process of finding information	Strongly disagree	37	13.5	22	4.0	3	1.9	46.216 (<0.001)
	Slightly disagree	128	46.7	236	43.4	65	40.9	
	Slightly agree	89	32.5	256	47.1	75	47.2	
	Strongly agree	20	7.3	30	5.5	16	10.1	

**Table 3:** Multinomial logistic regression analysis of factors associated with subjective health perception.

Variable		Medium vs. Good OR (95% CI)	*p*-Value	Poor vs. Good OR (95% CI)	*p*-Value
Sex Male (reference = female)		0.578 (0.413–0.807)	0.001	0.402 (0.247–0.655)	<0.001
Frequency of health information search lower frequency (reference = high frequency)		0.343 (0.166–0.709)	0.004	NS	–
Inconvenience in finding information (reference = Strongly agree)		NS	–	0.178 (0.038–0.842)	0.029
Understanding the doctor’s explanation (reference = Very easy)		NS	–	NS	–
Judging the reliability of media information (reference = Very easy)		3.118 (1.373–7.082)	0.007	6.213 (1.231–31.345)	0.027
Chronic disease	None (reference = ≥3)	NS	–	0.012 (0.002–0.061)	<0.001
	1 (reference = ≥3)	NS	–	0.074 (0.015–0.371)	0.002
Healthy eating habits (reference = very high effort)		2.806 (1.288–6.116)	0.009	5.544 (1.603–19.173)	0.007
Education (reference = University or higher)		NS; one category showed an unstable estimate	–	NS; one category showed an unstable estimate	–

Abbreviations: OR, odds ratio; CI, confidence interval; NS, not significant; Reference = good. The “good” subjective health perception group was used as the reference outcome category in the multinomial logistic regression analysis.

## Data Availability

The data used in this study were obtained from the Korea Institute for Health and Social Affairs (KIHASA). The dataset is available upon reasonable request from the corresponding institution.
